# Supplementary material for: MetaRibo-Seq measures translation in microbiomes
Source: Nat Commun. 2020 Jun 29;11:3268. doi: 10.1038/s41467-020-17081-z (PMC7324362; doi:10.1038/s41467-020-17081-z)
Supplement: Supplementary file 10 — Supplementary Data 7 [file 41467_2020_17081_MOESM10_ESM.zip › File2/Confidence_High_Taxonomy/9_out.krona.html]

Javascript must be enabled to view this page.

members
magnitude
magnitudeUnassigned
count
unassigned
taxon
rank

9\_out

6


SRS146888\_contig\_number\_contig-100\_28965.28966SRS147139\_contig\_number\_contig-100\_5880.178046
2

2
4
superkingdom

1239
4
phylum

class
4
186801

186802
order
4

species
4

SRS011084\_contig\_number\_3834SRS016495\_contig\_number\_12144SRS143876\_contig\_number\_37305SRS893253\_contig\_number\_contig-100\_8734.8735
1898207
